# The Role of Standing Variation in the Evolution of Weedines Traits in South Asian Weedy Rice (*Oryza* spp.)

**DOI:** 10.1534/g3.118.200605

**Published:** 2018-10-01

**Authors:** Zhongyun Huang, Shannon Kelly, Rika Matsuo, Lin-Feng Li, Yaling Li, Kenneth M. Olsen, Yulin Jia, Ana L. Caicedo

**Affiliations:** * Department of Biology, University of Massachusetts, Amherst, MA, USA, 01003; † Department of Biology, Washington University, St. Louis, MO 63130; ‡ Dale Bumpers National Rice Research Center, USDA-ARS, Stuttgart, AR 72160

**Keywords:** red rice, weed evolution, candidate genes, seed shattering, seed dormancy, Genomic Prediction, GenPred, Shared Data Resources

## Abstract

Weedy rice (*Oryza* spp.) is a problematic weed of cultivated rice (*O. sativa*) around the world. Recent studies have established multiple independent evolutionary origins of weedy rice, raising questions about the traits and genes that are essential for the evolution of this weed. Among world regions, South Asia stands out due to the heterogeneity of its weedy rice populations, which can be traced to at least three origins: two through de-domestication from distinct cultivated rice varieties, and one from local wild rice (*O. rufipogon*/*O. nivara*). Here we examine five traits considered typical of or advantageous to weedy rice in weedy, cultivated and wild rice samples from South Asia. We establish that convergence among all three weed groups occurs for easy seed shattering, red pericarp color, and compact plant architecture, suggesting that these traits are essential for weed success in the South Asian agricultural environment. A high degree of convergence for black hull color is also seen among weeds with wild ancestors and weeds evolved from the *aus* cultivated rice group. We also examine polymorphism in five known domestication candidate genes, and find that *Rc* and *Bh4* are associated with weed seed pericarp color and hull color, respectively, and weedy alleles segregate in the ancestral populations, as do alleles for the seed dormancy-linked gene *Sdr4*. The presence of a domestication related allele at the seed shattering locus, *sh4*, in weedy rice populations with cultivated ancestry supports a de-domestication origin for these weedy groups, and raises questions about the reacquisition of the shattering trait in these weedy populations. Our characterization of weedy rice phenotypes in South Asia and their associated candidate genes contribute to the emerging understanding of the mechanisms by which weedy rice evolves worldwide, suggesting that standing ancestral variation is often the source of weedy traits in independently evolved groups, and highlighting the reservoir of genetic variation that is present in cultivated varieties as well as in wild rice, and its potential for phenotypic evolution.

Agricultural weeds, defined as unwanted plants growing in agricultural environments (Monaco *et al.* 2002), are responsible for ∼30% annual reduction in crop productivity worldwide (Oerke 2006). This negative impact of weeds can be attributed to various traits, which allow them to adapt and be competitive in the agricultural environment. Examples of such traits in diverse species can include rapid growth, efficient seed dispersal and seed dormancy (Baker 1965), and can be considered to be part of an agricultural weed syndrome (Vigueira *et al.* 2013b). Weed syndrome traits have been found to evolve repeatedly and independently in different weed lineages (Vigueira *et al.* 2013a; Kane and Rieseberg 2008; Cui *et al.* 2016; Huang *et al.* 2017), and are examples of what is known as parallel or convergent evolution (Arendt and Reznick 2008).

Weedy rice *(Oryza spp*.), an aggressive unwanted weed of cultivated rice (*O. sativa*), is a type of agricultural weed displaying parallel evolution of weediness traits (Ziska *et al.* 2015). Weedy rice occurs worldwide, and studies have established multiple independent evolutionary origins of this weed from non-weedy backgrounds (Reagon *et al.* 2010; Huang *et al.* 2017; Vigueira *et al.*, 2017). Many typical traits of weedy rice resemble reversals to wild phenotypes. Despite diverse genetic backgrounds, red pericarp is a common feature of weedy rice, leading to the common name “red rice” (Gross *et al.* 2010; Cui *et al.* 2016). The fact that red pericarp is common likely reflects selection for dormancy in weedy rice seeds, since the gene which regulates proanthocyanidin pigment synthesis also pleiotropically regulates ABA regulated seed dormancy (Gu *et al.* 2011; Cui *et al.* 2016), and seed dormancy has been detected in several populations (Gianinetti and Cohn 2008; Ziska *et al.* 2015). Ease of seed shattering or dispersal has also been reported for several populations (Thurber *et al.* 2010; Zhu *et al.* 2012). The wealth of knowledge of candidate genes underlying many morphological traits in rice has recently made it possible to examine the origins of weedy traits in weedy rice populations (Gross *et al.* 2010; Vigueira *et al.* 2013b), as well as to examine the extent to which the same genes underlie convergent traits across weedy rice populations.

We recently characterized populations of weedy rice from South Asia (Huang *et al.* 2017), an area known for its great diversity of cultivated and wild *Oryzas* (Civáň *et al.* 2015; Garris *et al.* 2005; Huang *et al.* 2012; Zhu *et al.* 2007), that has also proven to contain a great diversity of weedy rice. Two weedy rice populations in this area trace their ancestry to cultivated rice varieties *aus* and *indica* through a process known as de-domestication (Huang *et al.* 2017). A third weedy population has evolved from local wild rice (the species complex known as *O. rufipogon/nivara*). Moreover, weeds presenting an admixture of all these backgrounds also occur (Huang *et al.* 2017). These South Asian weedy rice origins are also independent of the well-studied U.S. weedy rice populations de-domesticated from *aus* and *indica* varieties (Reagon *et al.* 2010; Huang *et al.* 2017). The diversity of weedy rice origins in a single world region raises questions about how each of these groups has acquired the traits that make them weedy.

To understand how weediness traits have evolved in each of the genetically distinct weedy populations of South Asia, we examine here the presence of iconic weedy rice traits in each of these populations. We additionally investigate the genetic mechanisms possibly underlying these traits by capitalizing on the extensive prior characterization of domestication trait candidate genes in cultivated rice, and deduce the evolutionary origins of alleles carried by weeds.

We first focus on pericarp pigmentation, as red pericarps have been proposed to be adaptive either by providing protection from abiotic or biotic stress (Ithal and Reddy 2004), and through association with seed dormancy (Gu *et al.* 2011). A red pericarp is also ubiquitous among wild rice, while during domestication most cultivated rice varieties were artificially selected to have white pericarps (Sweeney *et al.* 2006). The *Rc* gene, which codes for a basic helix-loop-helix protein in the proanthocyanin synthesis pathway, has been shown to underlie variation in pericarp color in *Oryza* (Sweeney *et al.* 2006).

The seed dormancy trait is also known to be affected by another gene in *Oryza*: *Sdr4* has been shown to be involved in differences in the degree of seed dormancy between the two cultivated rice subspecies: *japonica* and *indica* (Sugimoto *et al.* 2010). Seed dormancy is a trait common in wild rice but selected against during domestication, and is often reported for weedy rice; the capacity for dormancy over multiple seasons aids in propagation and persistence of weedy rice in crop field.

Another seed-related trait shown to be common in some weedy rice populations is black colored hulls (*e.g.*, Reagon *et al.* 2010). Wild rice tends to have dark hulls, while cultivated rice tends to have straw colored hulls. Dark hulls have been proposed as an adaptation aiding crypsis of the seed on the ground and avoidance of predators (Gu *et al.* 2011). The *Bh4* gene, encoding an amino acid transporter, is known to affect hull color (Zhu *et al.* 2011).

Easy seed shattering is one of the traits that most consistently differentiates weedy rice from cultivated rice, and is also a trait strongly selected against during domestication. Seed shattering is beneficial to weeds as it increases seed dispersal. Variation in a transcription factor encoding gene, *sh4*, is strongly associated with incomplete development of an abscission layer in the seed-pedicel junction and subsequent decrease of shattering during rice domestication Li *et al.* (2006).

A last trait we examine is plant architecture, as cultivated rice differs from wild rice in having narrower tiller angle and fewer tillers (Jin *et al.* 2008). In weedy rice, compact architectures have been proposed as adaptive, as they make the plants harder to distinguish from the crop, allowing evasion of removal efforts. Also plants with compact architectures are able to compete on equal footing for light (Ziska *et al.* 2015). The *PROG1* gene, encoding a zinc-finger nuclear transcription factor, has been implicated in this transition (Jin *et al.* 2008; Tan *et al.* 2008). The occurrence of *PROG1* domestication alleles has recently been implicated in the de-domestication origins of US weedy rice (Li *et al.* 2017).

Our goals were to: 1) determine which of these typical weedy traits characterize each of the known weedy rice populations occurring in South Asia; 2) investigate whether known candidate genes may explain the trait values found in each weedy rice population; and 3) determine whether weedy rice populations in South Asia have acquired weediness traits and alleles from standing variation in their ancestors, *de novo* mutation, or introgression from other sources. We further discuss the evolution of weediness traits in South Asian in context of recent discoveries of weedy rice evolution in other world regions.

## Material and Methods

### Plant material and phenotypic measurements

A panel of 101 *Oryza* accessions of types occurring in South Asia (including the countries Bangladesh, Myanmar, India, Nepal, Pakistan, and Sri Lanka) was used in this study. These included 35 South Asian weedy rice samples, for which predominant ancestries were previously characterized (Huang *et al.* 2017), comprising 15 *aus*-like, five *indica*-like, five wild-like and ten admixed weeds; admixed weeds are most often a mix of *indica* and wild backgrounds or *indica* and *aus* backgrounds (Huang *et al.* 2017). The panel also included *Oryza* groups potentially contributing to the genetic make-up of weedy rice; these were 44 cultivar varieties (15 *aus*, 13 *indica*, 11 *japonica* and five admixed cultivars), 18 wild ancestors of cultivated Asian rice (*O. rufipogon/O. nivara*), and four outgroup species (two *O. meridionalis* and two *O. barthii*) (Table S1).

Plant DNA was extracted as described in Huang *et al.* (2017) and used for sequencing of candidate genes (see below). We recorded seed traits for all 101 accessions from the original seed storage, with pericarp color classified as red or white, hull color classified as black or straw and awn as present of absent (Table S1).

We planted a subset of 44 accessions, with three replicates per accession, in a randomized design in two Conviron PGW36 growth chambers, under 11 hr day length with 25° temperature until 30 days after flowering. Due to limited chamber availability, the growth experiment was carried out in two stages. The first batch was planted on Feb 15, 2013 and the second on June 28, 2013. Tiller number and tiller angle were recorded at flowering. We divided tiller angle into three categories, <30°, 30-60° and 60-90°, as determined by the maximum tiller angle of the accession among three replicates. Seed shattering was measured as breaking tensile strength, using the method described in Thurber *et al.* (2010), which measures the weight needed to detach seeds from panicles 30 days after flowering.

### DNA sequencing and genotyping of candidate genes

Following the Agilent (Agilent, Santa Clara, CA) SureSelect Target Enrichment method (Gnirke *et al.* 2009) instructions, we designed baits of 120 bp length targeting the five candidate genes’ open reading frame (ORF). Additional baits were designed to target other known gene ORFs and gene fragments (∼1500 base pair (bp)) in upstream and downstream regions of each baited gene. A total of 128 loci were thus targeted and sequenced (Table S2). Baits were designed based on the rice reference genome (MSU 6.0 assembly; http://rice.plantbiology.msu.edu/). The SureSelect Target Enrichment method was applied to capture targeted genomic sequences in the *Oryza* panel, and Illumina Hi-seq 2500 sequencing was performed at the Whitehead Institute (Massachusetts Institute of Technology).

Raw sequencing reads were assessed for quality with FastQC software (http://www.bioinformatics.babraham.ac.uk/projects/fastqc/). The low quality reads were filtered using NGStoolkit (Patel and Jain 2012), keeping reads with depth >3 and mapping quality >30. Clean reads were mapped onto the MSU 6.0 reference genome using BWA (Li and Durbin 2009). SNP and indels were called with SAMtools (Li *et al.* 2009). A series of in-house Perl scripts were employed to convert the polymorphisms from variant call format (VCF) to FASTA format for sequence alignment. In the FASTA files, unmapped nucleotides were represented with N and low-quality nucleotides were represented with “?”.

Due to limitations of the SureSelect method to accurately detect moderate and large insertion/deletion (InDel) polymorphisms, we confirmed the presence of InDels with cleaved amplified polymorphisms (CAPS; Konieczny and Ausubel 1993) for two cases: the 14 bp InDel in exon 6 of *Rc* and the 22-bp InDel in exon 3 of *Bh4*. WebMap (http://pga.mgh.harvard.edu/web_apps/web_map_/start/) was used to create a map from the gene sequence files taken from the MSU database and Genbank. Restriction enzyme site complexity was set to greater than or equal to 5 residues in order to find an enzyme that would cut in the target region. Primer3 (http://frodo.wi.mit.edu/primer3/) was used to choose primers that would amplify the target region of the sequence.

The *Rc* gene was amplified using the primer pair Rc_4774_for (Sequence: tcaattcttgcatttcttttcca) and Rc_5299_rev (Sequence: acggctttatagaaatagagggagt). The *Bh4* gene was amplified with the primer pair Bh4_1_for (Sequence: caatctggtgcataatcagaatgga) and Bh4_1_rev primers (Sequence: cccgaagatcctgacgtagcag). The PCR profiles were set for one cycle of 95° of 3 min (*Rc*) and 30 sec (*Bh4*), 40 cycles of melting, annealing, and extension (95° for 1 min, 55° (*Rc*) and 59° (*Bh4*) for 30 sec, 68° for 1 min) and one cycle of 72° for 10 min. PCR products were digested with 0.5 ul of 20 units/ul restriction enzyme *Ban1* for *Rc* and *ApaLI* for *Bh4* by incubating at 37° for 60 min. The products of the digestion were analyzed using gel electrophoresis to identify insertion and deletion alleles in *Rc* and *Bh4*.

### Data Analysis

Sequence alignments among accessions were performed again for the 101 accessions using MUSCLE by EMBL-EBI (http://www.ebi.ac.uk/Tools/msa/muscle/). Aligned sequences were imported into BioEdit Ver.7.0.9 (Hall 1999) for manual refinement and localization of relevant polymorphisms. FASTA files of the alignments were exported and converted to PHYLIP format using PGDSpider software. We constructed haplotype trees for each candidate gene with the DNANJ tool implemented in the PHYLIP package (Felsenstein 1993) on CYVERSE (http://www.cyverse.org/), using Kimura 2-parameter distance model, with bootstrap values calculated via 1000 replicates, and two *O. meridionalis* samples (omd01 and omd02) designated as outgroups. MEGA and ITOL (http://itol.embl.de/) were used to visualize the trees.

The panel of accessions was separated into groups based on prior population structure analyses (Huang *et al.* 2017). For each candidate gene, information about un-translated regions, exons and introns were obtained from the Rice Genome Annotation Project database (http://rice.plantbiology.msu.edu/). Estimates of nucleotide diversity including Nei’s average pairwise nucleotide diversity (π) and Watterson’s estimator of theta (θ_w_) were calculated with in-house Perl scripts on each of the 128 loci for synonymous, non-synonymous, silent and all sites within each group. Tajima’s D (Tajima 1989) was also calculated for each group with in-house Perl scripts. Pairwise genetic distances between each group, assessed by F_ST_ values (Hudson *et al.* 1992), were calculated for seven population pairs, including three between weed groups and their ancestors (*aus*-like *vs.* *aus*, *indica*-like *vs.* *indica* and wild-like *vs.* wild), one between cultivars (*aus* *vs.* *indica*) and three among weed groups (*aus*-like *vs.* *indica*-like, *aus*-like *vs.* wild-like and *indica*-like *vs.* wild-like). Negative F_ST_ values were converted into zeros.

For all candidate genes, allele types occurring in each of the weed groups were compared to those of their non-weedy ancestors as determined in Huang *et al.* (2017). When sequenced haplotypes in weed groups were identical or very closely related to haplotypes found in ancestral groups, weedy alleles were considered to have been inherited from standing ancestral variation. In contrast, weed alleles most closely related to alleles belonging to a group different than that weed’s genomic ancestry, were taken as a possible sign of introgression. All candidate genes were examined for possible novel mutations in weed that could have an effect on gene function.

### Data availability

The authors state that all data necessary for confirming the conclusions presented in the article are represented fully within the article. Newly generated DNA sequences for candidate genes are available in GenBank (accessions MH771032 - MH771132, MH798903 - MH799306). DNA sequences for the 123 supplementary loci, as well as other supplementary material is available on figshare via the GSA portal. Supplementary tables contain genotype and phenotype of *Oryza* accessions included in the study, and genomic regions sequenced by target capture. Figure S1 contains the *PROG1* phylogenetic tree based on SNPs. Scripts used to obtain population statistics are available at Github: https://github.com/Zhongyun-Huang/G3-2018-200605. Supplemental material available at Figshare: https://doi.org/10.25387/g3.6853244.

## Results

### Phenotypic traits characterizing South Asian weedy rice groups

We had previously characterized several seed traits in a larger panel of South Asian weedy rice (Huang *et al.* 2017), and results for the subset of accessions characterized here confirm prior observations (Table 1, Table S1). Red pericarps are common in all groups of South Asian weedy rice (>60% of samples in weedy groups are red). This is in contrast to both progenitor cultivated rice groups, although they do show the presence of both pericarp types, but similar to wild rice (Table 1). Hull color varies among South Asian weedy rice, with *aus*-like, wild-like, and admixed weeds having primarily black hulls (>70%), while *indica*-like weeds have exclusively straw colored hulls (Table 1, Table S1). Cultivated groups tend to have straw colored hulls (>83% of our cultivated samples), and 83% of the wild rice samples we examined have black hulls.

**Table 1:** Categorical phenotypic traits in South Asian *Oryza* groups

		Pericarp		Hull		Tiller angle		
Group		red	white	black	straw	<30	30-60	60-90
Weedy rice	*aus*-like (15)	15 (100%)	0 (0%)	12 (80%)	3 (20%)	4	4	0
	*indica*-like (5)	3 (60%)	2 (40%)	0 (0%)	5 (100%)	2	2	0
	wild-like (5)	5 (100%)	0 (0%)	4 (80%)	1 (20%)	2	3	0
	admixed (10)	8 (80%)	2 (20%)	7 (70%)	3 (30%)	3	4	0
*aus* (14)		5 (36%)	9 (64%)	2 (17%)	12 (83%)	3	3	0
*indica* (13)		3 (23%)	10 (77%)	1 (8%)	12 (92%)	5	1	0
South Asian wild rice (18)		17 (94%)	1 (6%)	15 (83%)	3 (17%)	1	1	4

We additionally recorded the maximum tiller angle among replicates for each accession in our phenotyping study (Table S1). All South Asian weedy rice sub-groups have a relatively compact plant architecture, with angles <60 ^o^ (Table 1, Table S1). This is similar to the cultivated *aus* group, but not as compact as *indica*, which usually has tiller angles < 30°. As expected, wild rice has the most spread tillers, with the majority having angles between 60° and 90° (Table 1, Table S1). This result is consistent with previous observations on cultivars and wild rice (Jin *et al.* 2008).

We did not detect significant differences in tiller number among groups with the accession characterized for this study (*P* = 0.4), but trends confirmed prior significant observations from Huang *et al.* (2017), with higher tiller number occurring in wild rice and wild-like weeds (mean > 11), and lower numbers seen in cultivated *aus* and *indica* (mean < 7.71; Table 2). As previously reported (Huang *et al.* 2017), all weedy rice groups shatter easily, as does wild rice, and both cultivar-like weed groups shatter significantly more easily than their ancestors (*P* < 0.001; Table 2, Table S1).

**Table 2:** Quantitative phenotypic traits in Asian *Oryza* groups. Numbers in brackets beside means are standard deviations

		Tiller number^1^	Shattering^2^
South Asian weed (25)			
	*aus*-like (8)	8.44(3.35)	2.14(5.79)^d^
	*indica*-like (5)	11.11(6.68)	17.46(16.70)^bc^
	wild-like (5)	11.50(4.14)	8.36(19.05)^d^
	admixed (7)	7.57(3.95)	14.70(16.58)^c^
*aus* (7)		7.71(1.50)	25.61(17.92)^ab^
*indica* (6)		7.00(2.97)	35.96(5.49)^a^
South Asian wild rice (6)		11.44(7.55)	2.47(5.51)^d^
Kuskal-Wallis		0.4040086	**9.42E-08**

1 Chamber effect detected, only data from Chamber 1 plants were used in the analysis.

2 No chamber effect detected.

Taken together, our results suggest convergence among South Asian weedy rice groups in ease of shattering, red pericarp color and compact plant architecture. Additionally, convergence occurs among *aus*-like, wild-like and admixed weeds for black hull color.

### Patterns of polymorphism in Rc and Bh4

**Figure 1:**
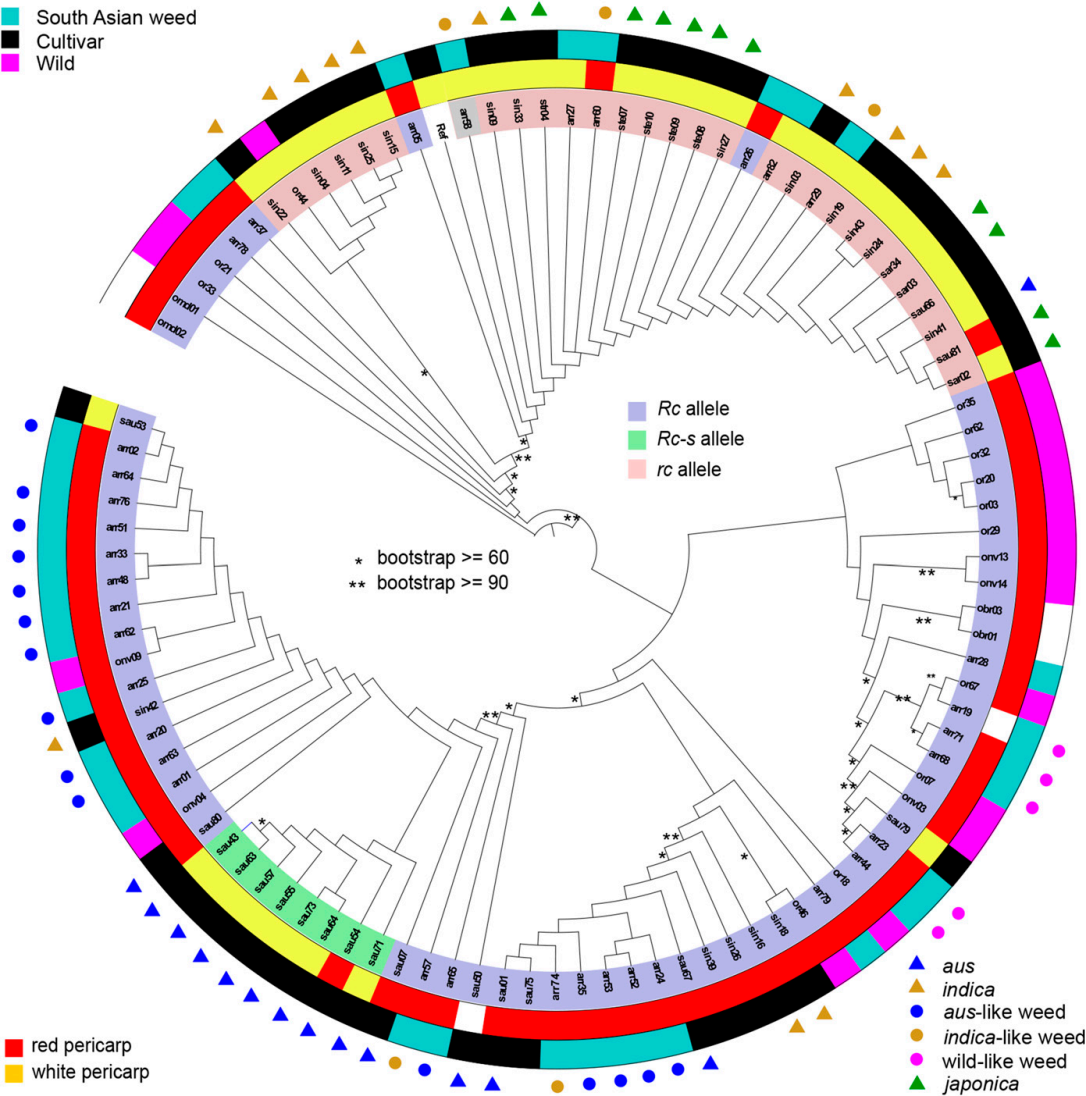
Neighbor joining (NJ) tree of *Rc* haplotypes. The color range indicates the three major alleles of the *Rc* gene; the inner ring indicates the colors of the pericarp. The outer ring and the bullets indicate *Oryza* groups, as labeled in the key. Unmarked weeds are admixed according to results of Huang *et al.* (2017). Asterisks on branches indicate bootstrap values as labeled.

*Rc* is considered a rice improvement gene contributing to the prevalence of white pericarps in cultivated rice, with the recessive white pericarp phenotypes caused by at least two known loss-of-function alleles: a 14-bp deletion (*rc*) in exon 6 or a point mutation from C to A (*Rc-s*) (Sweeney *et al.* 2006). We first carried out a phylogenetic analysis of the *Rc* coding region based solely on SNP information, which verified a single origin for the white pericarp conferring *Rc-s* allele in *aus* cultivars (Figure 1). We mapped on to the tree the presence and absence of the 14-bp deletion, allowing us to distinguish *Rc* from *rc* alleles (Figure 1). Our data supports a single origin of the *rc* allele, which is prevalent in, but not exclusive to, cultivars. The two *Rc* haplotypes observed in the *rc* clade may have come about by recombination of alleles without the deletion into an *rc* SNP background. Our data also support the ancestral status of *Rc* alleles, and their prevalence in wild rice. Further examination of the association between *Rc* allelic variation and pericarp color confirms that discrepancies between genotype and pericarp color are rare (six cases out of 95 accessions): most accessions with red pericarps carry the *Rc* allele and most accession with white pericarps carry the *rc* or *Rc-s* allele across most *Oryza* groups (Table 3, Table S1). This supports prior observations that *Rc* is the major gene underlying pericarp color, although other genes may also have effects.

**Table 3:** Pericarp color phenotype and *Rc* gene variation in *Oryza* accessions

	Pericarp color					
	Red			White		
Group	*Rc*	*rc*	*Rc-s*	*Rc*	*rc*	*Rc-s*
**Cultivars (43)**						
*aus* (14)	4	0	1	1	1	7
*indica* (13)	3	0	0	0	10	0
*japonica* (11)	0	1	0	1	9	0
admixed cutivar (5)	3	0	0	1	1	0
**South Asian weedy rice (34)**					
*aus*-like (15)	15	0	0	0	0	0
*indica*-like (5)	2	1	0	0	2	0
wild-like (4)	4	0	0	0	0	0
admixed (10)	8	0	0	0	2	0
**South Asian wild rice (18)**	17	0	0	0	1	0

As expected due to the high frequency of red pericarps, South Asian weeds predominantly carry *Rc* alleles (Figure 1, Table 3). Furthermore, there is phylogenetic structure in *Rc*-type alleles, such that South Asian wild-like weeds most closely resemble wild rice *Rc* haplotypes, and *aus*-like weeds tend to possess alleles that are most closely related to haplotypes present in *aus* cultivars, be they *Rc* or *Rc-s* alleles. *Indica*-like and admixed weeds possess alleles falling in both the *Rc* and *rc* clades, but in all cases similar alleles from ancestral groups are also observed. Thus, for both weeds of wild and cultivated descent, the red pericarp trait in all South Asian weedy rice groups is likely inherited directly from ancestors where this trait is either common (wild rice) or rare but present (cultivated rice).

**Figure 2:**
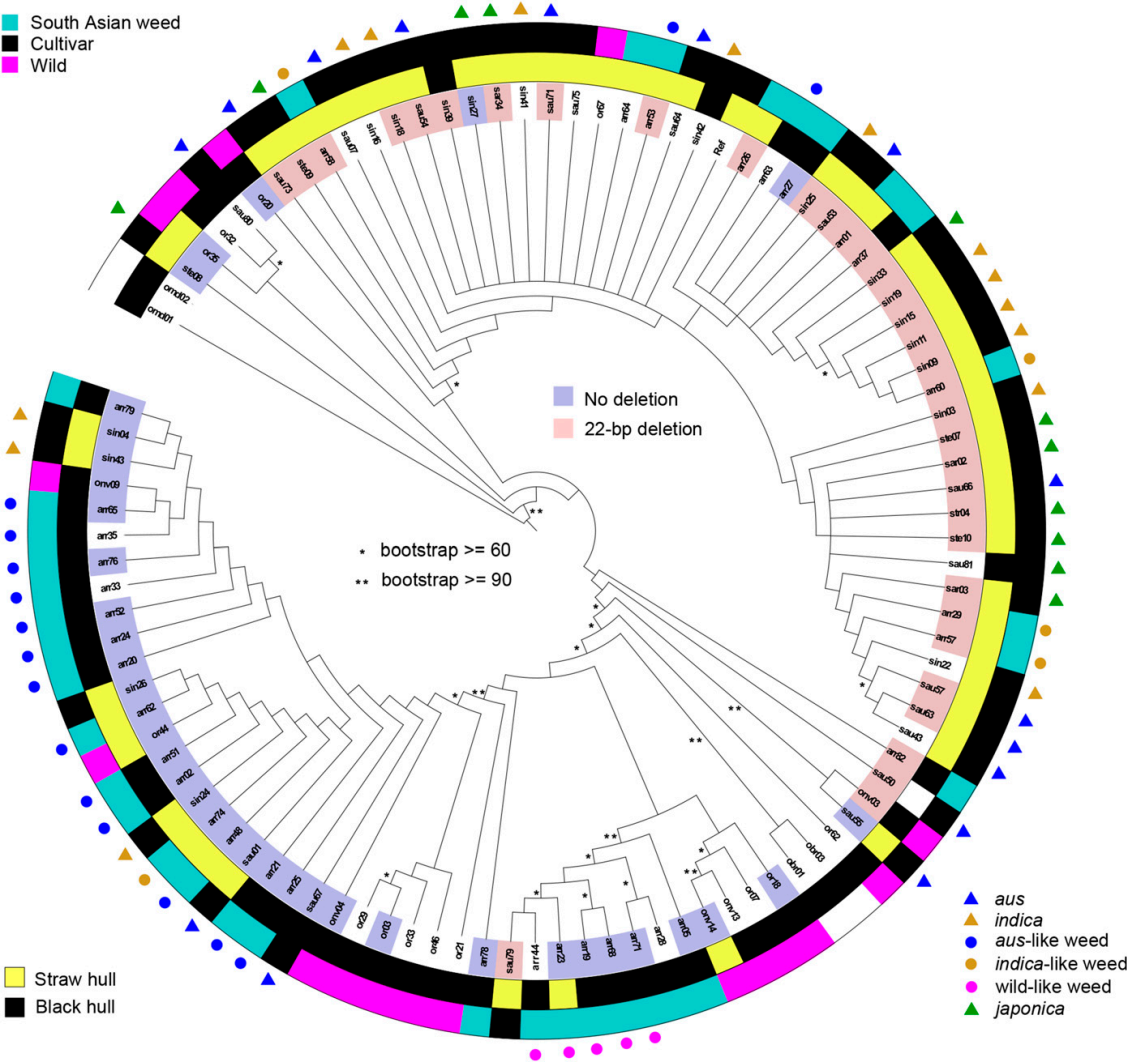
Neighbor joining (NJ) tree of *Bh4* haplotypes. The color range indicates the two major InDel alleles of *Bh4*; the inner ring indicates the hull color. The outer ring and the bullets indicate *Oryza* groups, as labeled in the key. Unmarked weeds are admixed according to results of Huang *et al.* (2017). Asterisks on branches indicate bootstrap values as labeled.

Like *Rc*, *Bh4* is considered a rice improvement gene that contributes to the straw colored hull phenotype that is common, but not fixed, in cultivated rice (Zhu *et al.* 2011). A 22-bp frame-shift deletion in exon 3 has been shown to be the dominant functional polymorphism causing a straw hull phenotype (Zhu *et al.* 2011). Again we carried out a phylogenetic analysis based solely on SNP data in the ORF of the gene, and mapped the presence or absence of the deletion onto the tree (Figure 2). Phylogenetic SNP analysis broadly supports a single origin for *Bh4* alleles carrying the 22 bp deletion, which are more common in crops, which tend to have straw-colored hulls, than in wild rice, which is predominantly black-hulled. A few deletion-carrying haplotypes occur outside the clade, as well as a few haplotypes without the deletion; recombination among alleles belonging to each clade could explain the rare lack of association between deletion status and *Bh4* SNP background.

Across *Oryza* groups, there is a strong association between hull color and the presence or absence of the *Bh4* deletion, with 75% of samples matching expected phenotype based on deletion status, but several exceptions were also observed (Table S3, Figure 2). For example three straw-hulled wild rice accessions and eight straw-hulled cultivars in our panel also have wild type alleles (Table S1, Table S3, Figure 2). This indicates that other loci can also affect hull color, as previously suggested (Kuriyama and Kudo 1967). In a previous survey of *Bh4* sequences, two other loss-of-function mutations were detected leading to straw hulls: a 1-bp frame shift deletion in exon 1 and a SNP in exon 3 that causes an early stop codon (Zhu *et al.* 2011), but neither was observed in our panel.

South Asian weeds of different origins carry *Bh4* haplotypes that primarily cluster with haplotypes of their ancestors (Figure 2). For example, wild-like weed alleles cluster together in the tree and seem to originate from a few closely related wild alleles. *Aus*-like and *indica*-like weeds with haplotypes carrying the 22 bp deletion cluster with various *aus* and *indica* haplotypes, and *aus*-like weeds lacking the 22 bp deletion also have *Bh4* haplotypes similar to these rarer haplotypes occurring in *aus* cultivars.

### Patterns of polymorphism in sh4

**Figure 3:**
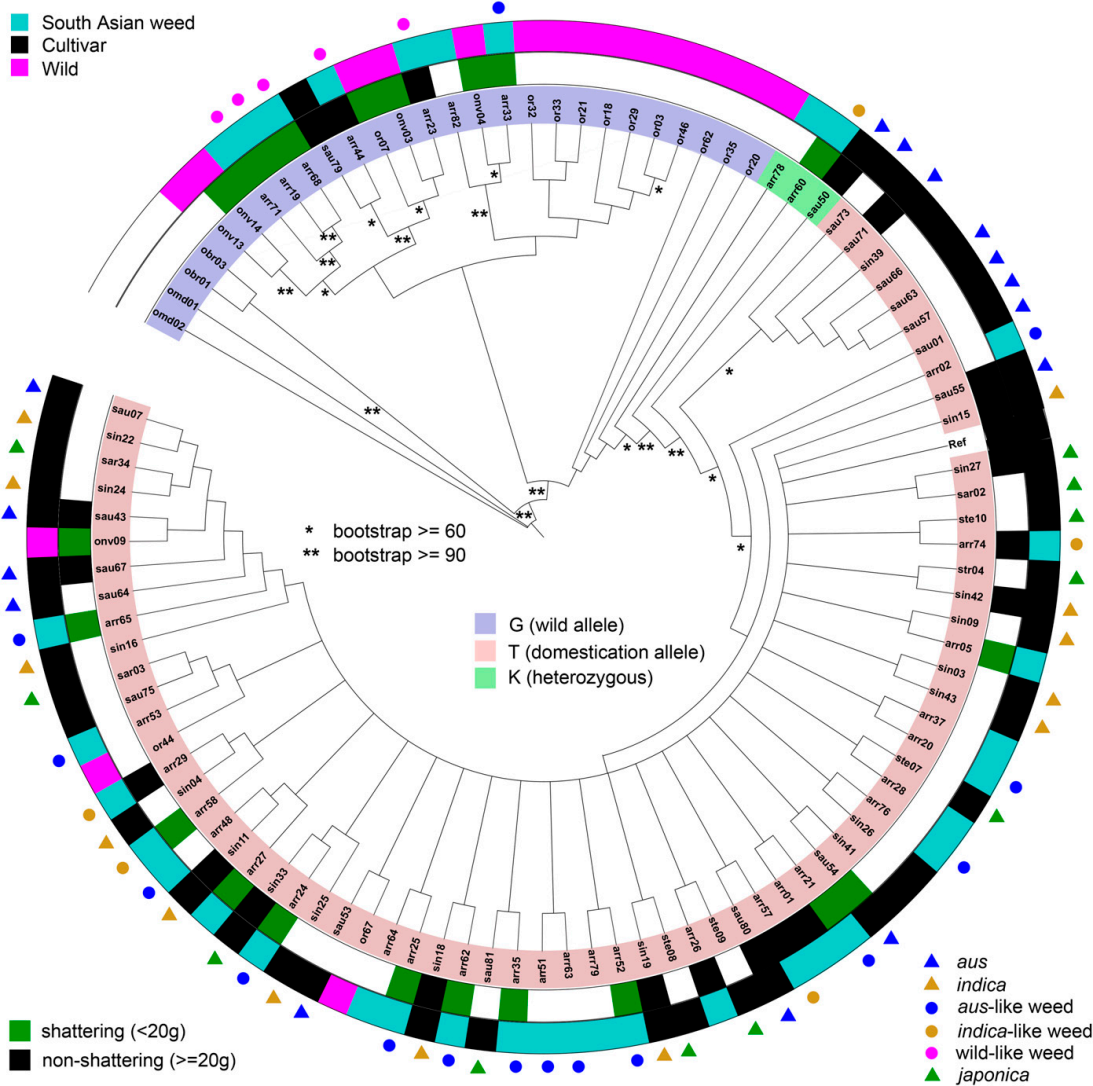
Neighbor joining (NJ) tree of *Sh4* haplotypes. The color range indicates the allele of the functional SNP; the inner ring indicates the shattering phenotype. The outer ring and the bullets indicate *Oryza* groups, as labeled in the key. Unmarked weeds are admixed according to results of Huang *et al.* (2017). Asterisks on branches indicate bootstrap values as labeled.

A single nonsynonymous G to T substitution in the first exon of *sh4* has been shown to lead to incomplete development of an abscission layer in the seed-pedicel junction in cultivated rice, and thus loss of seed shattering during domestication (Li *et al.* 2006). Our phylogenetic analysis of the *sh4* gene sequences obtained confirms the ancestral status of the G wild-type allele, and the prevalence of the derived T allele that is associated with loss of shattering in cultivated rice (Figure 3). South Asian weeds varied in the type of *sh4* alleles they possess. Wild-like weeds all bear the ancestral G allele, and *aus*-like and *indica*-like weeds (with only two exceptions) have the derived T allele, indicating that all weeds groups have inherited their *sh4* alleles from their respective ancestors. Given the near fixation of the T *sh4* allele during rice domestication (Li *et al.* 2006), these results support the origin of *indica*-like and *aus*-like weeds in South Asia through de-domestication (Huang *et al.* 2017).

Despite the fact that there is a strong correlation between seed shattering phenotype and *sh4* genotype in wild and cultivated rice (*i.e.*, all wild rice shatters to some degree and only one accession was found to carry the derived T allele; 93% of cultivars carry at least one T allele and 73% are non-shattering), this association breaks down when weedy rice groups are examined (Table S4, Table S1, Figure 3). The occurrence of easy shattering can easily be explained in wild-like weeds, as these all carry ancestral *sh4* alleles very similar to those found in their wild rice ancestors. However, *aus*-like and *indica*-like weeds also have a prevalent shattering phenotype (93% are medium or strong shattering; Table S4), yet all but one examined individual carried the derived domestication allele of *sh4* present in their cultivated ancestors. This suggests that these two weed-groups have evolved the shattering phenotype through an alternative genetic mechanism. This is similar to the case of U.S. weedy rice, which also arose through a process of de-domestication, and shatters despite fixation of the *sh4* T domestication allele (Thurber *et al.* 2010).

### Patterns of polymorphism in PROG1

The *PROG1* gene has been implicated in changes in plant architecture during rice domestication, with a single amino acid haplotype said to be present in cultivated rice, but multiple haplotypes present in wild ancestors (Jin *et al.* 2008; Tan *et al.* 2008). The occurrence of *PROG1* domestication alleles has also recently been implicated in the de-domestication origins of US weedy rice (Li *et al.* 2017). We used SNP variation in our *PROG1* sequences to construct a phylogenetic tree, and examined the distribution of haplotypes among the various *Oryza* groups. The causative mutation leading to the *prog1* allele associated with erect growth has not been isolated from the set of polymorphisms present in *PROG1*; however, wild rice has been shown to be polymorphic for PROG1 amino acid haplotypes, while most cultivated rice carries a single amino acid haplotype (Jin *et al.* 2008, Tan *et al.* 2008). We thus also examined the amino acid haplotypes present in our sequences (Figure S1). *PROG1* does not display much nucleotide or amino acid haplotypic diversity in our set of accessions, and, as previously reported, most cultivated rice of all varieties shares the same or similar alleles. Only four out of 44 cultivated rice accessions had alleles that were basal in the tree. In contrast, wild rice contains more different types of haplotypes, some of which are similar to those of cultivated rice, and some that are more basal. The bulk of South Asian weedy rice from all groups contains *PROG1* alleles that are similar or identical to the derived haplotypes predominating in cultivated rice. Weedy *PROG1* alleles can thus be explained through inheritance from standing variation in ancestral groups.

Our data did not yield an obvious association between tiller angle and *PROG1* alleles. All cultivated rice we measured has tiller angles that are less than 60°, and most accessions have similar *PROG1* alleles. This is true for weedy rice too, yet one wild rice accession with cultivated *PROG1* allele (or03) has broad tiller angle. Additionally, two wild-like weeds with very narrow tiller angels (<30°; arr23 and arr44) carry *PROG1* alleles that are closely related to an allele carried by a wild rice with a very broad angle (Figure S1). Thus, though *PROG1* allele identity in weeds can be explained by ancestral standing variation, our results suggest that tiller angle likely has other genetic determinants in our set of samples.

### Patterns of polymorphism in Sdr4

**Figure 4:**
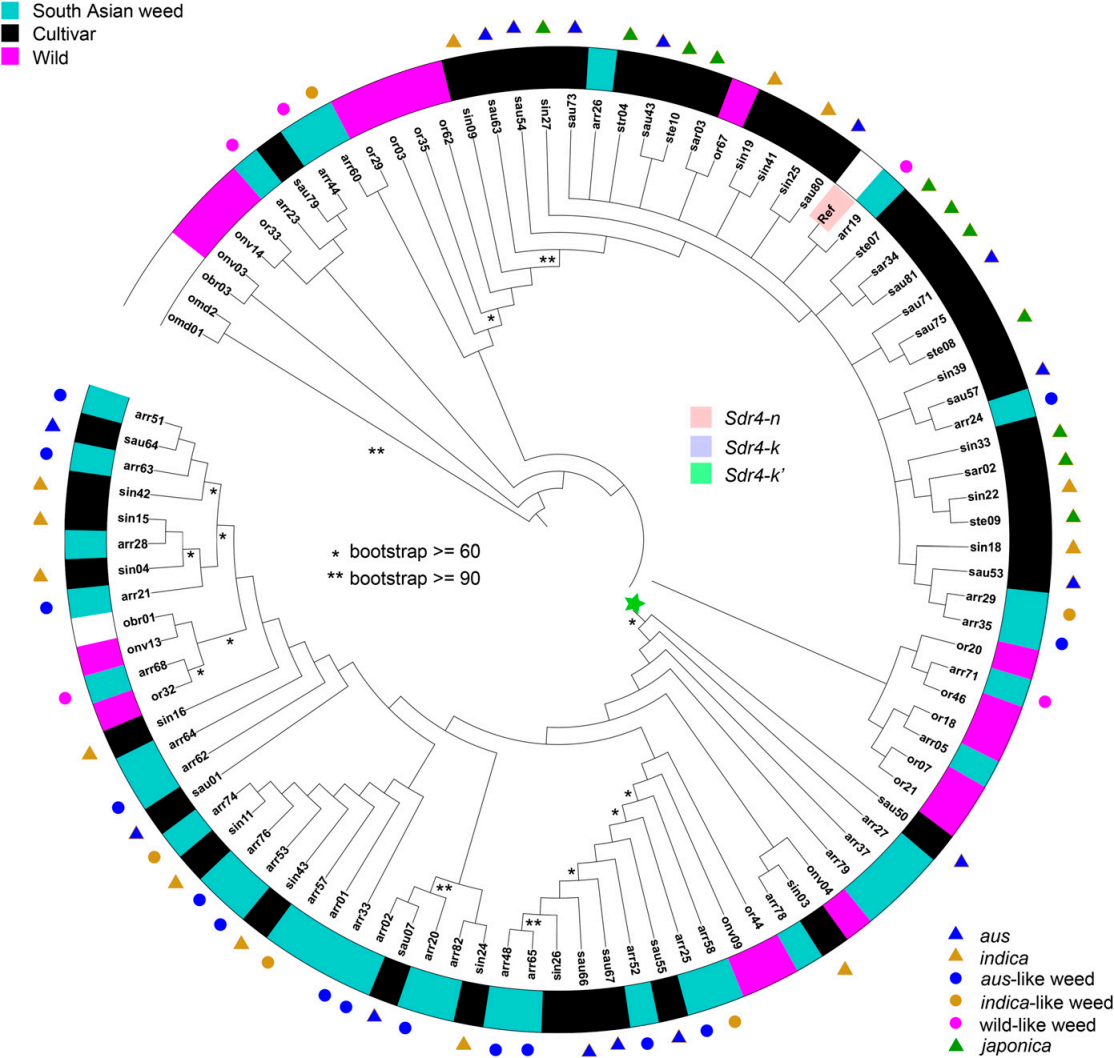
Neighbor joining (NJ) tree of *Sdr4* haplotypes. The color range indicates the three *Sdr4* haplotypes identified in Sugimoto *et al.* (2010). The ring and the bullets indicate *Oryza* groups, as labeled in the key. Unmarked weeds are admixed according to results of Huang *et al.* (2017). Asterisks on branches indicate bootstrap values as labeled.

The *Sdr4* locus has been implicated in the seed dormancy trait, with *Sdr4-k* alleles being common in wild rice (*O. rufipogon*) and present in some *indica* cultivar varieties, and *Sdr4-n* alleles conferring reduced dormancy in almost all *japonica* and some *indica* cultivated rice (Sugimoto *et al.* 2010). We used SNP information to construct a phylogenetic tree of *Sdr4*, and overlay information of allele type, based on the ten SNPs and one 3 bp indels that differentiate the reported *Sdr4-k* and *Sdr4-k* haplotypes, as well as the two SNPs and one 3 bp indel that differentiate *Sdr4-k* from its closely related, but rarer, *Sdr4-k*’ allele (Sugimoto *et al.* 2010). We detected many variations of *Sdr4*, with multiple haplotype that could not be classified as either *Sdr4-k* or *Sdr4-n* (Figure 4, Table S1). For cultivars, consistent with previous reports (Sugimoto *et al.* 2010), *japonica* accessions only have the *Sdr4-n* allele, while *Sdr4-n*, *Sdr4-k*, *Sdr4-k*’ alleles, as well as various other haplotypes occur in *aus* and *indica* accessions.

We found that very few weeds have inherited the *Sdr4-n* allele despite its occurrence in all cultivated and wild groups. Instead, most weeds carry haplotypes that are more similar to *Sdr4-k* and *k*’. Unfortunately, for this candidate gene we had no phenotypic data. However, if the previous association between *Sdr4* allele types and seed dormancy is taken at face value, our results suggest that most *aus*-like, *indica*-like, wild-like, and admixed South Asian weeds have some degree of seed dormancy conferred by the lack of *Sdr4-n* alleles. Moreover, although this trait may have been inherited from standing variation in cultivated and wild ancestors, it is significant that almost no weed derived from these groups has inherited the *Sdr4-n* variant that segregates in these groups, suggesting selection favoring *Sdr4-k* and similar alleles in South Asian weeds.

### Genetic diversity analyses

If weediness candidate genes were targeted by selection during weed evolution, a decrease in nucleotide diversity should be evident at these loci. We compared nucleotide diversity and Tajima’s D values at silent sites in candidate genes with those obtained from all 128 targeted loci as a representation of genome-wide averages for groups of weeds and their putative ancestors. Mean genome-wide nucleotide diversity is highest in wild rice and in wild-like weeds (Table 4). The two cultivar groups and their derived weeds have similar levels of diversity, suggesting that these de-domesticated weed groups have not experienced strong bottlenecks upon evolving from cultivated ancestors.

**Table 4:** Summary statistics on silent sites for candidate genes and all loci obtained with target-capture

		*Oryza* group					
Locus	Summary statistics	*aus*	*indica*	*aus*-like weed	*indica*-like weed	wild-like weed	wild
***Rc***	π	0.0025	0.0026	0.0021	0.0040	0.0029	0.0057
	θw	0.0029	0.0033	0.0018	0.0039	0.0027	0.0089
	Tajima’s D	−0.5243	−0.9636	0.6020	0.2043	0.4154	−1.5138
***Bh4***	π	0.0068	0.0092	0.0059	0.0096	0.0013	0.0141
	θw	0.0074	0.0077	0.0074	0.0115	0.0015	0.0130
	Tajima’s D	−0.2999	0.8084	−0.7779	−1.2185	−0.9726	0.3387
***sh4***	π	***0.00035***	***0***	**0.0009**	***0***	0.0090	0.0058
	θw	***0.00023***	***0***	**0.0021**	***0***	0.0083	0.0083
	Tajima’s D	1.1224	NA	**-2.124**	NA	0.6457	−1.2382
***PROG1***	π	***0***	0.0011	0.0010	0.0089	0.0089	0.0087
	θw	***0***	0.0024	0.0023	0.0106	0.0071	0.0107
	Tajima’s D	NA	−1.1491	−1.1594	−1.0485	1.4588	−0.6036
***Sdr4***	π	0.0018	0.0022	0.0018	0.0036	0.0036	0.0037
	θw	0.0011	0.0023	0.0033	0.0034	0.0035	0.0063
	Tajima’s D	1.3759	−0.1269	−1.3165	0.2431	0.2431	−1.3434
**All 128 genes**	π	0.0041	0.0040	0.0044	0.0048	0.0062	0.0090
	θw	0.0040	0.0043	0.0044	0.0049	0.0060	0.0105
	Tajima’s D	−0.0211	−0.2047	−0.0885	−0.1591	0.2740	−0.8180

Nucleotide diversity (π or θw) of individual gene <0.0005.

Tajima’s D >2 or < -2.

Most candidate genes surveyed have levels of nucleotide diversity that are unremarkable compared to genomic averages, not exceeding two standard deviations from the mean. *Sh4* stands out for its very low levels of diversity in *aus*, *indica* and *indica*-like groups, and this is consistent with selection for reduced shattering during the domestication process of cultivar groups (Li 2006; Konishi *et al.* 2006; Thurber *et al.* 2010). Low *sh4* nucleotide diversity in *indica*-like weeds can be attributed to the lack of diversity at this gene in ancestral *indica*. Other candidate genes showing extremely low levels of nucleotide diversity are *PROG1* and *sh4* in the *aus* group, which suggests selection on some aspect of plant architecture in *aus* (Table 4).

Weedy groups derived from crops did not show bottleneck evidence on a genome-wide level (Table S5), but wild-like weeds on average possess about 60% of their ancestral diversity. Loss of diversity in wild-like weeds is particularly evident in *Rc* and *Bh4* suggesting possible selection on these pericarp and hull coloration genes in wild-like weeds. In contrast, crop-derived weeds show remarkable increases in diversity for *PROG1* compared to their ancestors. This could be due to strong selection on cultivars for domesticated plant architecture, coupled with release of selection during de-domestication of the weeds. However, it is notable that weeds resembled crops in their predominant tiller angles (Table 1). A similar situation is seen for *sh4* in *aus*-like weeds, where high diversity could be due to release from selection.

Genome-wide F_ST_ values for the three weed-ancestor comparisons show that *indica*-like weeds have negligible amounts of differentiation from *indica* cultivars (F_ST_ = 0.014), while differentiation between *aus*-like weeds and *aus* (F_ST_ = 0.172) and wild-like weeds and wild rice (F_ST_ = 0.165) is moderate (Table 5). Among candidate genes, *Bh4* stands out by exceeding genome-wide F_ST_ values by more than two standard deviations (SD) from the mean between *aus*-like weeds and *aus*. This is consistent with the higher frequency of black hulls observed in *aus*-like weeds compared to their cultivated ancestors and could indicate selection favoring black-hull *Bh4* haplotypes in weeds (Table 1).

**Table 5:** Pairwise F_ST_ between weed groups and ancestors for each of the 15 candidate genes (**Hudson *et al.* 1992**)

	*aus*-like *vs. aus*	*indica*-like *vs. indica*	wild-like *vs.* wild
***Rc***	0.0123	0.0000	0.2917
***Bh4***	0.6362	0.0000	0.3067
***Sh4***	0.0884	NA	0.1047
***PROG1***	0.1364	0.0124	0.2072
***Sdr4***	0.2279	0.0000	0.1386
**All 128 genes**	0.1717	0.0142	0.1654

## Discussion

We had previously investigated the evolutionary origins of weedy rice in South Asia, an area harboring great cultivated and wild *Oryza* diversity (Huang *et al.* 2017). Weedy rice in this region has a highly heterogeneous genetic background, more so than many other world regions (Reagon *et al.* 2010; Vigueira *et al.* 2017; Li *et al.* 2017), with contributions from two cultivated rice varieties (*aus* and *indica*) and from the wild ancestor of domesticated rice. Despite these very diverse origins, our current study finds that weedy rice groups in South Asia share the presence of several iconic weediness traits, supporting the concept of an agricultural weed syndrome (Vigueira *et al.* 2013b), and helping define the suite of traits that are most likely to be adaptive in weedy rice.

**Figure 5:**
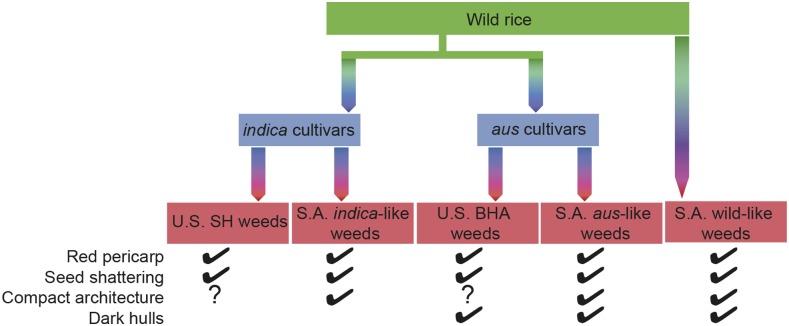
Summary figure of known convergent traits among the South Asian weedy rice groups in this study and previously studied U.S. weedy rice groups. Featured weedy groups have been shown in Huang *et al.* (2017) to have all evolved independently. Convergence in red pericarps may be indicative of convergence in seed dormancy, which was not directly evaluated.

Of the traits examined, the greatest convergence among all three weed groups in South Asia occurred for easy seed shattering, red pericarp color, and compact plant architecture (Table 1; Table 2; Figure 1; Figure 3; Figure S1; Figure 5), suggesting that these traits are advantageous in the South Asian agricultural environment. Convergence among the wild and cultivar derived weedy rice groups is striking, given that for some of these traits weeds do not resemble their ancestors. For example, red pericarps and shattering phenotypes do not predominate in cultivars but are common in *aus*-like and *indica*-like weeds; likewise, wild rice tends to have a spreading architecture, yet wild-like weeds tend to be more compact. Convergence for seed shattering and red pericarp also occurs with weed groups in other parts of the world such as the U.S. (Thurber *et al.* 2010; Gross *et al.* 2010; Figure 5) and Malaysia (Song *et al.* 2014; Cui *et al.* 2016), supporting the idea that these are important traits in weedy rice evolution. Unfortunately, there is not enough information in the literature to gauge if compact plant architecture is also common in other weed groups. Our data suggest then, that among the traits surveyed, seed dormancy potentially related to red pericarps, and seed shattering are the most essential weediness traits in weedy rice worldwide.

Given separate origins, how have weedy groups in South Asia acquired convergent weedy traits? Our survey of *PROG1* as a candidate gene for tiller angle is inconclusive, but our candidate genes for pericarp color and seed shattering suggest differing genetic mechanisms. Most weedy accessions with red pericarps carry wild-type *Rc* alleles that can explain the phenotype and were likely inherited from variation present in their ancestral groups (Figure 1). The similar nucleotide diversity in the *Rc* gene for all cultivar and weed groups, as well as the low pairwise F_ST_ between weed and ancestor, also serve as evidence of the inheritance of ancestral alleles (Table 4; Table 5). The very occasional discrepancies between pericarp color and *Rc* alleles (Figure 1, Table 3) indicate that other genes or mutations may affect pericarp color, but the *Rc* genotype in weeds is mostly sufficient to explain weed phenotype. Consistent with origins for ancestral standing variation, there are no strong signatures of selection observed for this gene in weed groups (Table 3).

A similar situation to pericarp color and *Rc* may be occurring with seed dormancy and its candidate gene *Sdr4*. We unfortunately do not have seed dormancy phenotypic data for our weedy populations, but seed dormancy has often been considered a characteristic weedy trait (Sugimoto *et al.* 2010). The lack of dormancy-associated *Sdr4-n* alleles in any weed population, while easily explained through inheritance from ancestral standing variation, suggests possible convergence in seed dormancy among all weed groups possibly mediated through the evolutionary advantage conferred by maintenance of the dormancy trait.

Convergence of seed shattering in South Asian weeds seems to have occurred in a different manner. In our South Asian panel, every wild-like weed has the same ancestral wild type *sh4* allele as wild rice that has been shown to be associated with seed shattering (Li *et al.* 2006), thus *sh4* inheritance from ancestors can explain the shattering phenotype in wild-like weeds. In contrast, almost all cultivar-like weeds have the derived *sh4* allele common in crop rice (Table S4; Figure 3) that has been implicated in loss of shattering during domestication. Thus, although inheritance from cultivated ancestors explains the *sh4* allele present in *indica* and *aus*-like weeds, this allele cannot explain the shattering phenotype. This is similar to what has been observed for US weedy rice (Thurber *et al.* 2010), where a novel genetic mechanism must account for seed shattering. Whether there is convergence in this novel mechanism between South Asian cultivar-derived weeds and any of the US groups is unknown, and we cannot yet draw conclusions about the roles of standing variation *vs.* new mutations in the evolution of shattering in these groups. The high levels of nucleotide diversity of *aus*-like weed in *sh4* compared to *aus* cultivars, and the extremely negative Tajima’s D (Tables 4; Table S5), suggest that *aus*-like weed may be undergoing a release in selection for this gene.

While not common to all weedy groups in South Asia, a high degree of convergence for black hull color occurs among wild-like, admixed and *aus*-like weeds. This trait is also convergent with one group (BHA) of US weedy rice (Reagon *et al.* 2010). The fact that dark hulls and awns are present, but not prevalent in the *aus* ancestors of both BHA and *aus*-like South Asian weeds (Table S3), suggests there may be some adaptive value to these traits such as resistance to pests and predators. However, these cannot be considered essential weedy traits, as weedy rice groups that lack them nevertheless thrive around the world (Ziska *et al.* 2015; Song *et al.* 2014). Both wild-like and *aus*-like weeds likely inherited these phenotypes from variation present in ancestral groups. In the case of hull color, this is supported by *Bh4* variation (Figure 2), with wild-like weeds carrying *Bh4* alleles similar to wild rice, and *aus*-like weeds carrying alleles similar to the *aus* in our panel with no-deletion alleles. While the black hull phenotype is rare in the *aus* in our panel, a slightly higher frequency has been previously reported for a wider set of *aus* surveyed (Huang *et al.* 2017). Surprisingly, although *aus*-like weeds likely inherited *Bh4* alleles from a small subset of *aus* cultivars with black hulls, both groups show similar nucleotide diversity at *Bh4* (Table 4; Table S5), but very large differentiation as measured by F_ST_ (Table 5). This is compatible with a scenario whereby *aus*-like weeds were de-domesticated from *aus* cultivars early in the domestication process, before the majority of *aus* cultivars underwent selection for straw hulls with the 22-bp *Bh4* deletion (Vigueira *et al.* 2013a).

Our characterization of weedy rice phenotypes in South Asia and the associated candidate genes contribute to the emerging understanding of the mechanisms by which weedy rice evolves worldwide. Whether evolving from crop ancestors, as inferred for BHA and SH weeds in the US and *aus*-like weeds and *indica*-like weeds in South Asia, or evolving from wild ancestors, such as the wild-like weeds of South Asia, standing variation present in the non-weedy ancestors often seems a sufficient explanation for the origin of weedy rice traits. Thus, for none of the genes surveyed, even those that underlie traits where weeds diverge from their ancestors, is introgression required to explain the alleles present in weedy rice (the only exception being the unknown genetic mechanism for shattering in cultivar-related weeds). The fact that cultivated rice varieties, which have been shown to have gone through domestication bottlenecks and loss of diversity (Caicedo *et al.* 2007), as well as wild rice, which is not *a priori* adapted to the agricultural environment, nevertheless harbor the necessary standing variation (*i.e.*, allelic diversity) to give rise to troublesome weeds, may help explain the prevalence of the weedy rice problem worldwide. The possibility of continued repeated evolution of weedy rice presents an interesting conundrum to usual calls for maintenance of diversity in crop species.
